# Regional metabolic and network changes in Meige syndrome

**DOI:** 10.1038/s41598-021-95333-8

**Published:** 2021-08-03

**Authors:** Jiayu Liu, Lei Li, Yuan Li, Qian Wang, Ruen Liu, Hu Ding

**Affiliations:** 1 Department of Neurosurgery, Peking University People’s Hospital, 11th Xizhimen South St, Beijing, 100044 China; 2 Department of Nuclear Medicine, Zhuhai People’s Hospital (Zhuhai Hospital Affiliated with Jinan University), No. 79 Kangning Road, Xiangzhou District, Zhuhai, 519000 Guangdong China; 3 Department of Nuclear Medicine, Peking University People’s Hospital, 11th Xizhimen South St., Beijing, 100044 China

**Keywords:** Neuroscience, Computational neuroscience, Neurological disorders

## Abstract

To contribute to the understanding of the aetiology and pathogenesis of Meige syndrome, the metabolic networks of patients with Meige syndrome were investigated using 18F-fluoro-D-glucose positron emission tomography (18F-FDG-PET) imaging of cerebral glucose metabolism. Fifty right-handed and unmedicated primary Meige syndrome patients enrolled between September 2017 and September 2020 at the Department of Neurosurgery, Peking University People’s Hospital, and 50 age- and sex-matched healthy control subjects participated in the study. Metabolic connectivity and graph theory analysis were used to investigate metabolic network differences based on 18F-FDG-PET images. Glucose hypometabolism was detected in the left internal globus pallidus and parietal lobe, right frontal lobe and postcentral gyrus, and bilateral thalamus and cerebellum of patients with Meige syndrome. Clustering coefficients (Cps) (density threshold: 16–28%; P < 0.05) and shortest path lengths (Lps) (density threshold: 10–15%; P < 0.05) were higher in Meige syndrome patients than in healthy controls. Small-worldness was lower in Meige syndrome patients than in healthy controls, and centrality was significantly lower in the right superior occipital gyrus and pallidum and higher in the right thalamus. Hypometabolism in the globus pallidus and thalamus may indicate basal ganglia-thalamocortical motor circuit abnormalities as a pathogenic mechanism of Meige syndrome, providing a possible explanation for the efficacy of deep brain stimulation (DBS) in improving symptoms. Meige syndrome patients had abnormal small-world properties. Centrality changes in the right pallidus and thalamus verified the important roles of these regions in the pathogenesis of Meige syndrome.

## Introduction

Meige syndrome, also known as blepharospasm-oromandibular dystonia syndrome, was first described by and subsequently named after French neurologist Henri Meige in 1910^1^. It is a rare segmental cranial-neck dystonia. This disease starts insidiously and develops slowly. It mainly involves the muscles of the eyelid, mouth, jaw and neck and seriously affects the work and life of patients. The aetiology and pathogenesis of this disease have not been fully defined.

According to recent studies, the loss of inhibition at different levels in the central nervous system may lead to Meige syndrome. In patients with dystonia, the striatum's inhibitory output to the pallidum is enhanced, resulting in decreases in the average discharge rate and output of the pallidum as well as excessive enhancement of cortical motor function, leading to excessive movement^2^. Dresel et al.^3^ investigated cortical function changes in patients with simple blepharospasm and Meige syndrome compared with normal controls with simple blepharospasm using functional magnetic resonance imaging (fMRI) and found that cerebral cortical inhibitory function was reduced in patients with Meige syndrome. Furthermore, Feiwell et al.^4^, using positron emission tomography imaging (PET) in 7 patients with blepharospasm and 7 normal subjects, found that compared with normal subjects, patients had significantly reduced blood flow in primary sensorimotor areas following facial vibration, suggesting that abnormal sensorimotor processing in dystonia patients, including abnormal sensory afferent and central processing, can lead to muscle cramps.

However, not all of the subjects in the above study were patients with Meige syndrome, and the sample size was small. Therefore, it is difficult to draw strong conclusions about the pathogenesis of Meige syndrome, which is a rare disease. A previous voxel-based morphological study by our research team involving 46 patients with Meige syndrome showed that the precuneus is involved in the development of Meige syndrome, which indicated that patients with Meige syndrome may have abnormal integration at the cortical level^5^. However, we did not observe involvement of the basal ganglia and motor cortex in the pathophysiology of the disorder. The precuneus has been shown to have extensive connections with cortical and subcortical structures, such as the basal ganglia^6^. We suspect that although functional changes in brain regions such as the basal ganglia are necessary for the development and maintenance of dystonia, they do not necessarily result in anatomical changes^5^. Therefore, further studies, such as fMRI or PET studies, are necessary to further explore the pathogenesis of Meige syndrome.

Previous studies examining functional brain diseases have focused mostly on measuring structural, functional and metabolic changes in specific brain regions. This method of studying specific brain regions is direct and simple but fails to reflect how the brain as a complex system realizes its functions. In recent years, complex network methods have been widely used to investigate many brain diseases^7^. Among the many brain network analysis methods, graph theory analysis can provide many indexes for quantifying network characteristics, such as the clustering coefficient (Cp), shortest path length (Lp) and small-world index^8^. These indicators can change when there is damage to the brain. Structural magnetic resonance imaging (MRI) and fMRI^9^, PET imaging^10^, electroencephalography (EEG)^11^, and magnetoencephalography (MEG)^12^ have been used in the study of human brain networks. As a traditional functional imaging method, ^18^F fluoro-D-glucose (FDG)-PET can directly detect changes in glucose metabolism to reflect the activity of brain tissue, offering a special advantage over other imaging modes^13^. Therefore, we propose to examine metabolic network properties to identify pathogenic features in Meige syndrome. To our knowledge, this study is the first to explore the characteristics of metabolic network features in patients with Meige syndrome.

The goal of the current study was to investigate metabolic characteristics and networks in Meige syndrome using FDG-PET. We mainly attempted to determine which brain regions are affected in terms of glucose metabolism and to assess the changes in network parameters in patients with Meige syndrome relative to healthy controls.

## Results

### Baseline characteristics

A total of 50 Meige syndrome patients (25 males; 25 females) were included, and the age of the participants ranged from 39 to 76 years (52.06 ± 8.71). In the healthy control group, a total of 50 individuals (25 males; 25 females) were included, and the average age was 53.52 ± 8.01 years. Baseline characteristics (sex and age) did not significantly differ between the two groups. Furthermore, fasting blood glucose did not significantly differ between the healthy control and Meige syndrome patient groups (5.40 ± 0.67 mmol/L vs. 5.30 ± 0.41 mmol/L, respectively). Disease duration in the patients with Meige syndrome was 6.48 ± 5.67 years (Table 1).

**Table 1:** Characteristics of the subjects.

	Meige syndrome (min to max)	Healthy control (min to max)	p
Gender (male)	25	25	1.000
Age (years)	52.06 ± 8.71 (36.00 to 73.00)	53.52 ± 8.01 (36.00 to 75.00)	0.385
Fasting blood glucose (mmol/L)	5.40 ± 0.67 (4.30 to 5.80)	5.30 ± 0.41 (4.10 to 5.80)	0.749
Duration of disease (years)	6.48 ± 5.67 (1.50 to 28.00)	–	–

### Glucose hypometabolism in Meige syndrome

**Figure 1:**
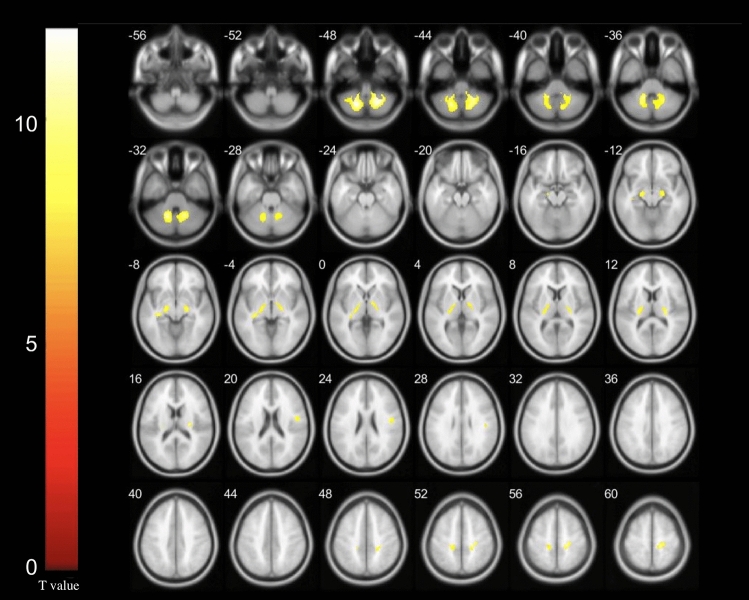
The regions with decreased glucose metabolism in Meige syndrome (P < 0.001). Cluster size > 50 voxels. The colour bar indicates the T-value.

After controlling for the effects of age, sex, and glucose level, multiple significant differences in local brain glucose metabolism rate were revealed between the patients with Meige syndrome and controls. In the patients with Meige syndrome, we found glucose hypometabolism in the left hemisphere in the internal globus pallidus and parietal lobe and in the right hemisphere in the frontal lobe and postcentral gyrus. In addition, in the patients with Meige syndrome, the bilateral thalamus and cerebellum were found to have glucose hypometabolism (Table 2, Fig. 1).

**Table 2:** The regions with decreased glucose metabolism in Meige syndrome.

AAL regions	Volume (voxels)	T	Coordinates		
			x	y	z
Internal globus pallidus (left)	405	6.27	− 32	− 28	− 6
Frontal lobe (right)	85	6.18	48	− 8	22
Postcentral gyrus (right)	249	6.97	22	− 28	54
Parietal lobe (left)	58	5.98	− 18	− 32	50
Bilateral thalamus	271	6.60	26	− 20	14
Bilateral cerebellum	1121	7.32	22	− 60	− 48

Coordinates x, y, z refer to the anatomical location of peak voxel defined by the Montreal Neurological Institute space. All regions significant on voxel level P < 0.001.

### Small-world topology of the functional networks

**Figure 2:**
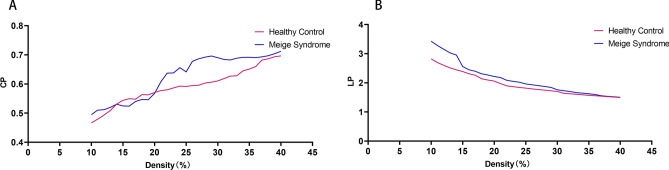
The clustering coefficient (Cp) and shortest path length (Lp) in Meige syndrome patients and healthy controls. **(A)** The Cp values of patients with Meige syndrome and healthy controls. P < 0.05 (significant at 16–28%). **(B)** The Lp values of patients with Meige syndrome and healthy controls. P < 0.05 (significant at 10–15%).

The Cp in the Meige syndrome group was higher than that in the healthy control group when the density threshold was 16–28%, and the difference between the two groups was statistically significant (P < 0.05) (Fig. 2A). The shortest Lp in the Meige syndrome group was higher than that in the healthy control group. When the density threshold was within the range of 10–15%, the differences in Lp value between the two groups were statistically significant (P < 0.05) (Fig. 2B).

**Figure 3:**
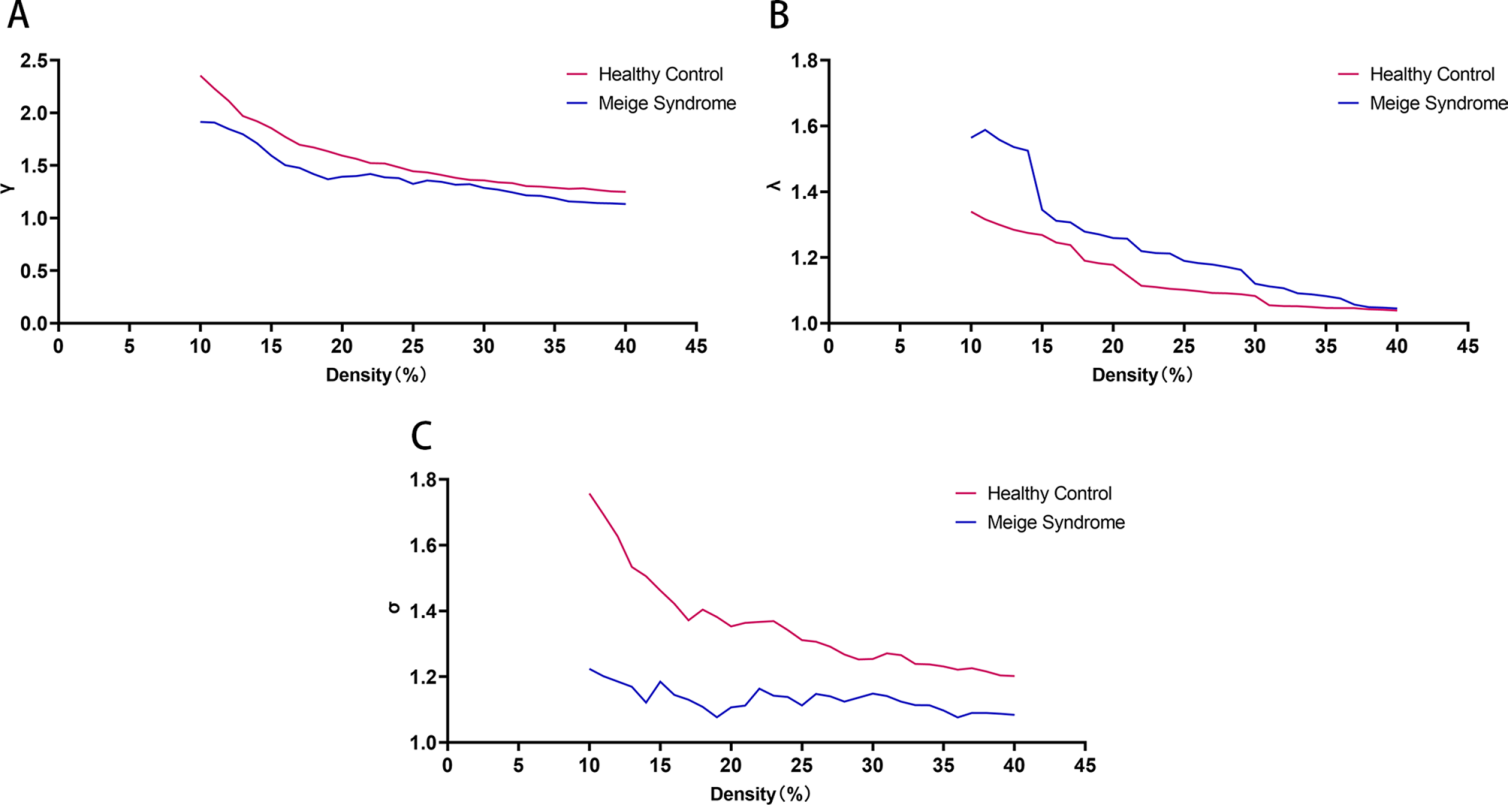
Normalized cluster coefficient (γ), normalized characteristic path length (λ), and small-world index (σ) in Meige syndrome patients and healthy controls. **(A)** The γ values of patients with Meige syndrome and healthy controls. **(B)** The λ values of patients with Meige syndrome and healthy controls. P < 0.05 (significant at 10–14%). **(C)** The σ values of patients with Meige syndrome and healthy controls. P < 0.05 (significant at 10–25%).

In the cerebral metabolic networks, across conditions with varying density thresholds (10–40%), the standardized Cp values were greater than 1 (γ >  > 1) in both groups, and the standardized shortest Lp values were ≈1 (λ ∼ 1) in both the Meige syndrome group and the healthy control group. Therefore, there was a small-world index (σ) > 1 in both groups. However, across the whole threshold range, the small-world characteristics in the Meige syndrome group were all lower than those in the healthy control group. Our study showed that the normalized Cp (γ) values were similar between the groups (Fig. 3A) and that the normalized characteristic Lp (λ) values were significantly greater in the Meige syndrome group than in the healthy control group (density threshold: 10–14%; Fig. 3B); therefore, the small-world index (σ) was significantly lower in the Meige syndrome group than in the healthy control group (density thresholds: 10–25%; Fig. 3C) (P < 0.05).

### Distribution of the hub regions and altered nodal centrality in Meige syndrome

**Figure 4:**
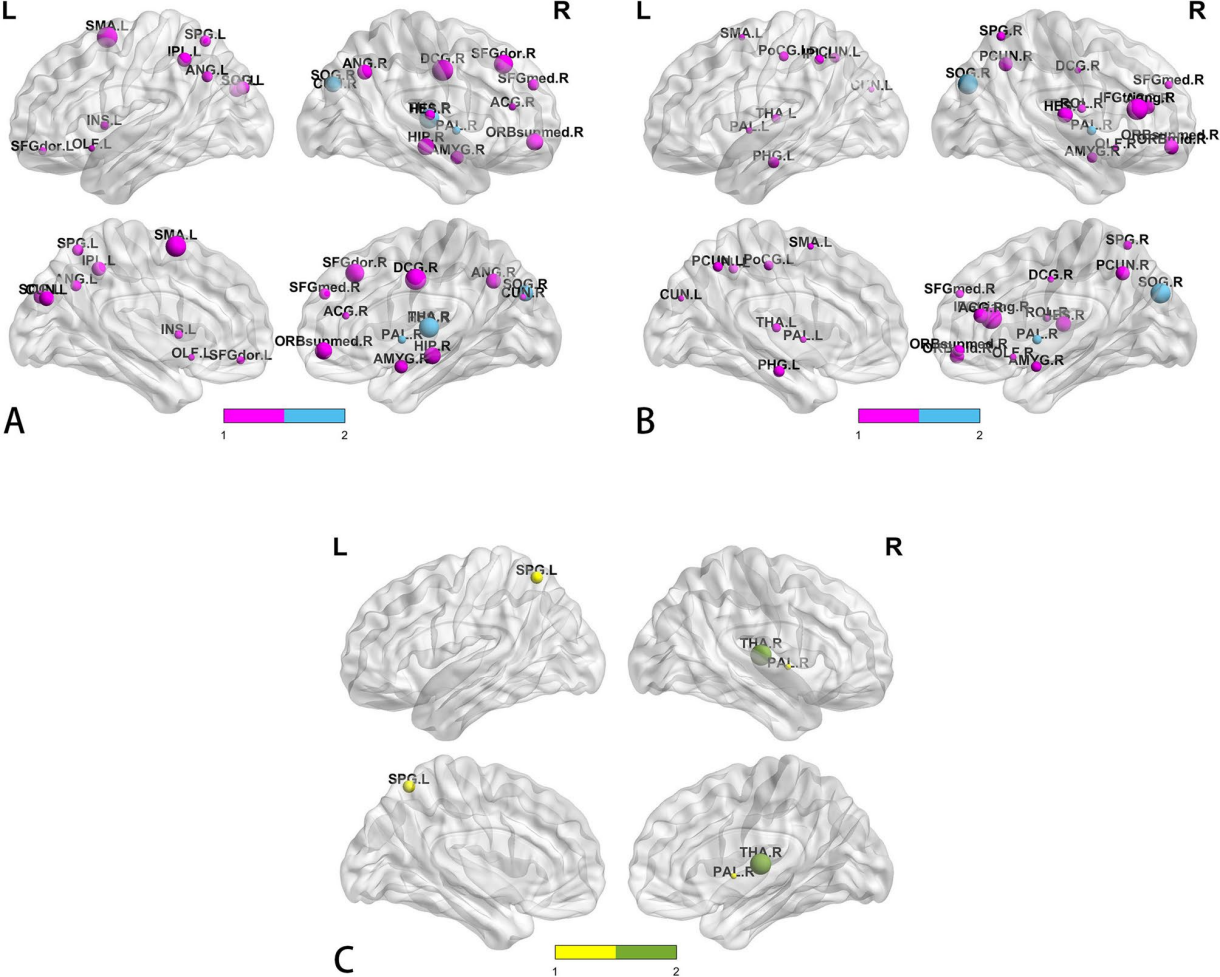
**(A)** The hub regions in patients with Meige syndrome. **(B)** The hub regions in healthy controls. **(C)** Differences in betweenness centrality between Meige syndrome patients and healthy controls (P < 0.05). The blue spheres represent the common hub regions. Sphere diameter indicates normalized betweenness centrality (B(i)); in this case, B(i) > 2.0. The yellow spheres represent betweenness centrality with significant decreases (two hub regions), and the green spheres represent betweenness centrality with significant increases (one hub region) in patients with Meige syndrome compared with healthy controls. Hubs were visualized with BrainNet Viewer (http://www.nitrc.org/projects/bnv).

We defined hub regions by calculating the normalized betweenness centrality (B(i)) of the 90 brain regions defined by the automated anatomical labelling (AAL) template. Both the Meige syndrome group (Table 3) and the healthy control group had 22 hub regions (Table 4). The two groups shared 12 hub regions (Fig. 4A,B, blue spherical area). We used a nonparametric permutation test with 1000 repetitions and identified three hub regions with significant differences between the two groups (P < 0.05; Fig. 4C). The centrality was significantly reduced in the right superior occipital gyrus and the right pallidum (Fig. 4C, yellow sphere area) and significantly increased in the right thalamus (Fig. 4C, green sphere area) in the Meige syndrome group compared with the healthy control group.

**Table 3:** The hubs of the functional network in the Meige syndrome group.

Region (Meige syndrome)	Abbreviations	b*i*
Superior frontal gyrus, dorsolateral (right)	SFGdor.R	7.31
Superior frontal gyrus, orbital part (left)	ORBsup.L	3.07
**Supplementary motor area (left)**	**SMA.L**	**8.94**
Olfactory cortex (left)	OLF.L	2.25
**Superior frontal gyrus, medial (right)**	**SFGmed.R**	**6.12**
**Superior frontal gyrus, medial orbital part (right)**	**ORBsupmed.R**	**4.38**
Insula (left)	INS.L	3.36
**Anterior cingulate and paracingulate gyri (right)**	**ACG.R**	**2.65**
**Median cingulate and paracingulate gyri (right)'**	**DCG.R**	**7.24**
Hippocampus (right)	HIP.R	4.87
**Amygdala (right)**	**AMYG.R**	**3.41**
**Cuneus (left)**	**CUN.L**	**4.66**
Cuneus (right)	CUN.R	2.01
Superior occipital gyrus (left)	SOG.L	9.09
**Superior occipital gyrus (right)**	**SOG.R**	**5.08**
**Superior parietal gyrus (right)**	**SPG.R**	**6.04**
**Inferior parietal, but supramarginal and angular gyri (left)**	**IPL.L**	**4.39**
Angular gyrus (left)	ANG.L	5.69
Angular gyrus (right)	ANG.R	4.50
**Lenticular nucleus, pallidum (right)'**	**PAL.R**	**3.20**
Thalamus(right)	THA.R	9.90
**Heschl gyrus (right)**	**HES.R**	**4.95**

This table list the hub regions (bi > 1.5) in the functional network of healthy control group. The common hub regions for both groups are indicated by bold. bi denotes the normalized betweenness centrality of the region i.

**Table 4:** The hubs of the functional network in the healthy control group.

Region (healthy control)	Abbreviations	b*i*
Middle frontal gyrus, orbital part (right)	ORBmid.R	6.53
Inferior frontal gyrus, triangular part (right)	IFGtriang.R	9.73
Rolandic operculum (right)	ROL.R	3.68
**Supplementary motor area (left)**	**SMA.L**	**2.22**
Olfactory (right)	OLF.R	2.55
**Superior frontal gyrus, medial (right)**	**SFGmed.R**	**3.63**
**Superior frontal gyrus, medial orbital part (right)**	**ORBsupmed.R**	**8.03**
**Anterior cingulate and paracingulate gyri (right)**	**ACG.R**	**7.19**
**Median cingulate and paracingulate gyri (right)'**	**DCG.R**	**2.31**
Parahippocampal gyrus (left)	PHG.L	7.68
**Amygdala(right)**	**AMYG.R**	**6.36**
**Cuneus(left)**	**CUN.L**	**2.03**
**Superior occipital gyrus (right)**	**SOG.R**	**9.91**
Postcentral gyrus (left)	PoCG.L	5.22
**Superior parietal gyrus (right)**	**SPG.R**	**4.32**
**Inferior parietal, but supramarginal and angular gyri (left)**	**IPL.L**	**4.98**
Paracentral_Lobule (left)	PCL.L	5.31
Paracentral_Lobule (right)	PCL.R	5.38
Lenticular nucleus, pallidum (left)	PAL.L	2.32
**Lenticular nucleus, pallidum (right)**	**PAL.R**	**4.73**
Thalamus (left)	THA.L	4.22
**Heschl gyrus (right)**	**HES.R**	**4.21**

This table list the hub regions (bi > 1.5) in the functional network of Meige syndrome group. The common hub regions for both groups are indicated by bold. bi denotes the normalized betweenness centrality of the region i.

## Discussion

This is the largest study to investigate the changes in cerebral glucose metabolism in Meige syndrome and the first to explore the changes in the topological properties of the cerebral glucose metabolism network in these patients. Our findings are as follows: (1) There were changes in brain metabolism in patients with Meige syndrome, with decreased metabolism in the internal globus pallidus and parietal lobe in the left hemisphere and in the frontal lobe and postcentral gyrus in the right hemisphere. The thalamus and cerebellum of patients with Meige syndrome were found to have glucose hypometabolism in both hemispheres. (2) The Meige syndrome group was found to have a significantly lower small-world index, higher Cp, and higher characteristic Lp than the healthy control group. (3) The spatial distribution of hub regions and nodal centrality were different between the Meige syndrome group and the control group. Centrality was significantly reduced in the right superior occipital gyrus and the right pallidum and increased in the right thalamus in patients with Meige syndrome.

Abnormal basal ganglia function has long been considered closely related to dystonia^14^. The function of the basal ganglia is to integrate sensory, motor, emotional and other cortical information and transmit the integrated information back to the cortex, and this process plays an important role in maintaining normal cortical excitability. Previous reports have shown that injury to the globus pallidus can lead to secondary dystonia^15^. However, in our previous voxel-based morphological study, we did not observe the involvement of the basal ganglia and motor cortex in the pathophysiology of the disorder in Meige syndrome^5^. Our experiences with stereotaxic surgeries have revealed that deep brain stimulation (DBS) of the internal globus pallidus or subthalamic nucleus alleviates Meige syndrome^16,17^. The basal ganglia might be a key structure in the mechanisms underlying Meige syndrome. We suspect that function in these brain regions might change during the development and maintenance of Meige syndrome. In this study, we found that metabolism was decreased in the left interna globus pallidus in the patients with Meige syndrome, which confirmed our previous hypothesis. A neuronal activity study^18^ demonstrated that in dystonia, the mean discharge rate of neurons in the internal globus pallidus is considerably reduced, and the discharge is irregular. We believe that a similar pathogenesis of patients with Meige syndrome was identified in this study. The improvement of Meige syndrome following inhibition within the internal globus pallidus might result from an interruption of the abnormal pattern of pallidal output^19^. In this study, only the left pallidum showed reduced metabolism. Although such basal ganglia asymmetry has been reported in subjects with dystonia of the hand, further studies are needed to clarify the causes of unilateral changes in metabolism in the pallidum^20^. Furthermore, glucose hypometabolism in both hemispheres of the thalamus was found in patients with Meige syndrome in this study. This finding suggests that the pathogenic mechanisms underlying Meige syndrome may be related to abnormal basal ganglia-thalamocortical motor circuits. Functional disruptions of the basal ganglia-thalamocortical motor circuit lead to errors in movement scaling and focusing^21^.

We also found a metabolic decrease in the posterior central gyrus in the right hemisphere in Meige syndrome patients. People with dystonia usually exhibit the "sensory trick" phenomenon^22^, which may be related to deficiencies in sensorimotor integration in dystonia, so we believe that this disorder of higher sensory processing is closely related to the occurrence of Meige symptoms. The results of the present study are consistent with those of Martino et al.^23^ and Piccinin et al.^24^, who found that grey matter volume in the sensory cortex was reduced in patients with primary blepharospasm compared with controls. In addition, metabolic reductions in the bilateral cerebellar hemispheres in patients with Meige syndrome were found in the present study. Delmaire et al.'s voxel-based morphometry (VBM) study^25^ revealed reduced grey matter in the cerebellar cortex of subjects with handwriting spasms. Argyelan et al.’s diffusion tensor imaging (DTI) study^26^ showed that patients with dystonia had decreased fractional anisotropy (FA) values of fibre bundles in the cerebellar thalamocortical motor circuit. Considering these previous findings and the reductions in bilateral thalamic metabolism found in this study, we believe that the cerebellar thalamocortical motor circuit is involved in the pathogenesis of Meige syndrome. Furthermore, in the present study, metabolism was decreased in the right frontal lobe of patients with Meige syndrome. The frontal lobe is an important component of emotion-related pathways^27^. Meige syndrome patients always have depression and other emotional disorders. We believe that the reduction in frontal metabolism shown in this study was mainly related to nonmotor symptoms in these patients, which is consistent with the results of our previous VBM study^5^.

The results of this study showed that PET functional brain networks in both the Meige syndrome and healthy control groups had small-world properties. The brain is a complex network of multiple brain regions, and an increasing number of studies have shown that the human brain has efficient small-world properties^28^. In a study on the functional network of patients with Alzheimer's disease and mild cognitive impairment, it was found that the PET brain functional networks of healthy people, patients with Alzheimer's disease and subjects with mild cognitive impairment all conformed to small-world properties^29^. In this study, both the healthy controls and Meige syndrome patients had networks with small-world properties, indicating that even in the state of disease, human brains can effectively integrate and process all kinds of information to meet the needs of daily life. However, the Meige syndrome patients had a lower small-world index (σ) than healthy controls (Fig. 3C). In our subsequent analysis, we found that the differences in small-world properties between patients with Meige syndrome and healthy controls were significant. Our results showed that compared with healthy controls, Meige syndrome patients had significantly higher Cp and characteristic Lp values (Fig. 2A,B). Shorter Lps can ensure more efficient information transmission and integration between remote brain regions^30^; the increased Lp in patients with Meige syndrome indicated that the collaborative working efficiency of remote brain regions was slowed in these patients. Cp reflects the local information processing rate and fault tolerance of the network^31^. The increased Cp of the network in patients with Meige syndrome indicated that the processing efficiency of the brain for local information was improved, which might compensate for the decrease in collaborative working efficiency between remote brain regions. We believe that altered brain small-world properties might serve as diagnostic indicators in patients with Meige syndrome^32^. However, the complex network associated with Meige syndrome is still in the preliminary stage of exploration, and the identification of specific network markers of Meige syndrome warrants further research.

Betweenness centrality (B(i)) describes the centrality of node i in the entire network, which is a parameter defined based on the perspective of network information flow. When B(i) is greater than 2.0, the region represented by node i is considered a hub region in the network^33^. Figure 4A,B show the hub regions of the brain networks in the healthy control group and the Meige syndrome group, respectively. Our study found that in both groups, most of the hub regions were located in the associative cortex and the paralimbic system, which play important roles in the functional integration of different brain regions^34^. There were 12 core regions present in both patients with Meige syndrome and healthy controls (Fig. 4A,B, blue spherical area), which may indicate that these regions are less susceptible than other regions to the remodelling process of the brain network with Meige syndrome. Moreover, our results showed that compared with the healthy control group, the Meige syndrome group had levels of centrality in the right superior occipital gyrus and the right pallidum that were significantly reduced (Fig. 4C, yellow sphere area). The hub region defined by node centrality mainly reflects the importance of a specific region in the whole network and integrates the information of the whole network. Therefore, a decrease in the core region centrality represents a decrease in the importance of the region in the whole network. The occipital lobe is located just behind the parietal lobe and is primarily associated with vision, in which the input comes from the lateral geniculate part of the thalamus^35^. Frequent blepharospasm in patients with Meige syndrome results in difficulty opening the eyes and visual impairment in severe cases^36^. Therefore, the decrease in B(i) in the superior occipital gyrus may be related to visual function changes in patients with Meige syndrome. Regarding the metabolic differences described above, we found a decrease in B(i) on the left side of the internal globus pallidus in patients relative to controls but no change on the right side. The B(i) change in the right pallidus verified the important role of this region in the pathogenesis of Meige syndrome, as mentioned above. Although the metabolism of the right globus pallidus was not decreased, its ability to integrate information in the whole-brain network was decreased, which may be related to the onset of Meige syndrome. Our results also showed that centrality was significantly increased in the right thalamus (Fig. 4C, green sphere area) of patients with Meige syndrome. Since decreased B(i) in hub core regions may lead to increased B(i) in others^37^, the increased B(i) of functional networks in the right thalamus in patients with Meige syndrome may represent a compensatory mechanism for decreased centrality in the right superior occipital gyrus and pallidum^38^.

## Conclusion

In this study, we investigated the metabolic characteristics and topological properties of brain networks in Meige syndrome patients. The combination of FDG-PET and graph theory analysis provided novel insights into the pathogenesis of Meige syndrome. First, we demonstrated hypometabolism of the globus pallidus and thalamus in Meige syndrome, indicating that the pathogenic mechanism of Meige syndrome may be related to abnormalities in the basal ganglia-thalamocortical motor circuit and providing a possible explanation for the efficacy of DBS in Meige syndrome. Second, compared with healthy controls, Meige syndrome patients had abnormal small-world properties. The B(i) changes in the right pallidus and thalamus verified the important roles of these regions in the pathogenesis of Meige syndrome. In conclusion, our study provides a potential pathophysiological basis for Meige syndrome and imaging evidence for brain network abnormalities in Meige syndrome.

This study has some deficiencies. First, although this study is the largest study of Meige syndrome to date, due to the low incidence of this disorder, the sample size is small. Second, due to the insufficient sample size, we were unable to analyse patients with different types of Meige syndrome (oromandibular dystonia, blepharospasm and combined oromandibular dystonia and blepharospasm). Third, each smoothing method has its limitations. Although we used mask to control image spill over in the standardized step, the ROIs are not big enough to avoid risk of spill over due to the limit spatial resolution in this study. Our PET resolution is 5 mm, and FWHM is 8 mm. The resolution of PET is not enough, which may lead to partial spill over. Further high-resolution PET-related studies are needed. In addition, the use of standard ROIs (AAL atlas) might not allow correction for partial volume effects that may be pronounced in small structures, such as the pallidum, under investigation. In future studies, we will collect MRI data from all studied Meige syndrome patients and use a subject-specific atlas derived from individual structural T1-MRI to perform correction. Finally, this study investigated only the whole-brain network of patients with Meige syndrome and not local brain networks. Further studies are necessary to better elucidate the pathogenesis of Meige syndrome.

## Methods

### Participants

Data from 50 right-handed and unmedicated patients with primary Meige syndrome were collected between September 2017 and September 2020 at the Department of Neurosurgery, Peking University People’s Hospital. The diagnostic criteria were mainly based on blepharospasm, oromandibular dystonia and cervical dystonia, increased blink rates and other symptoms^39^. Primary Meige syndrome was diagnosed by an experienced neurologist, Ruen Liu. The exclusion criteria were as follows: 1. neurological diseases other than Meige syndrome, such as Parkinson's disease or severe cognitive dysfunction; 2. serious mental illness, such as schizophrenia; 3. metabolic diseases such as diabetes, hyperthyroidism or hypothyroidism; 4. positive urine toxicology or pregnancy tests before any scan; 5. psychotropic medication use within the past 2 months; and 6. other serious systemic diseases, such as severe organic heart disease, severe lung, liver and kidney dysfunction, or coagulation dysfunction. Fifty age- and sex-matched healthy control subjects also participated in the study.

Written informed consent was obtained from each participant, and the study was approved by the institutional review board of Peking University People’s Hospital. All methods were carried out in accordance with relevant guidelines and regulations.

### PET scanning

All patients fasted for at least 6 h before PET/CT imaging, and the blood glucose of the patients was controlled to below 6.0 mmol/L. ^18^F-FDG (provided by Atom High-Tech Co., Ltd., Beijing, China) was injected intravenously at 5.55 MBq/kg (0.15 mCi/kg)^40^. After resting for 60 min in a dark, quiet environment, each patient lay in a supine position on the examination bed and then underwent head PET scans for 8 min. The spatial resolution of the scanner was 4.2-mm full width at half maximum (FWHM) in the axial, sagittal or coronal plane. The PET scan used the 3-dimensional (3D) mode.

### Preprocessing of PET data

PET images were analysed using SPM8 (https://www.fil.ion.ucl.ac.uk/spm) in the MATLAB R2016a programming environment (The MathWorks, Natick, MA). The PET data were preprocessed before analysis. First, spatial normalization was performed using the standard template of the Montreal Neurological Institute (MNI). Second, smoothing was performed by convolution using an isotropic Gaussian kernel with an 8-mm FWHM to increase the signal-to-noise ratio.

### Metabolic analysis

With the assistance of SPM8, the smoothed images were subjected whole-brain voxelwise statistical comparison (independent two-sample t-test) between patients with Meige syndrome and normal controls (healthy controls = 50, patients with Meige syndrome = 50). Normalization of FDG images by whole-brain signals was performed prior to the voxelwise comparison for the metabolic analysis. Then, we controlled for age, sex, and glucose variability. The output of the comparison was an SPM t-Map showing clusters of statistically significant voxels. With the help of xjView software (version 9.6, https://www.alive learn.net/xjView), MNI spatial localization and image display of brain regions with local differences in brain glucose metabolism were carried out.

### Network analysis

The whole-brain network construction process based on graph theory was mainly divided into four steps. First, AAL was used to segment the preprocessed brain PET images into 90 regions of interest (ROIs; 45 in each hemisphere except cerebellum) representing nodes^9^. Then, to obtain the average glucose metabolism value in each brain region, we normalized the glucose metabolism values in the 90 ROIs in the brain (dividing the mean glucose metabolism value in each ROI by that in the whole brain). Third, to remove the influences of age, sex and blood glucose on glucose metabolism from the ROIs, we introduced these variables into a linear regression model to obtain the corresponding regression residuals. Finally, Pearson correlation analyses of the residuals were used to construct the corresponding 90 × 90 correlation coefficient matrices. In this study, “1” represents an edge with a correlation connection, and “0” represents an edge without a correlation connection^41^. Based on a previous study^42^, we thresholded each correlation matrix over a wide range of densities (10–40% with a 1% increment) and then estimated the properties of the resulting graphs at each threshold value. In the present study, the lowest density where the largest component size was 90 was 10%. A density range larger than 10% ensured that every nodal pair in both graphs had a connecting path and minimized the number of false-positive paths^43^.

- 1. Cp: Cp is an important parameter that measures the degree of network clustering^8^ and is the average value of the Cps of all nodes in the network. The Cp C_i_ is a parameter reflecting the connection density of the local subnet around node i, which is related to the local efficiency of the network and quantifies the compensation capacity of the local network when node i is lost.
- 2. Characteristic Lp: The shortest Lp is an important parameter that measures the efficiency of network information flow^44^. It is the average value of the shortest Lp between any two nodes in the network. A lower shortest past corresponds to faster information exchange in the network and lower resource consumption of the network system.
- 3. “Small-world” network: To quantify the “small-world” characteristics of these networks, a random network was used as a reference. If the network being studied has a larger Cp and a higher estimated shortest Lp relative to those of a random network, then the network is a “small-world” network^42^. Some researchers have proposed unifying the two metrics into one metric, the small-world index (σ), σ = γ/λ, to measure the “small-world” characteristics of the network^45^. (normalized Cp (γ) = Cp real/Cp rand ≫ l and normalized characteristic Lp (λ) = Lp real/Lp rand ≈1, where “rand” denotes a random network and “real” denotes a real network.)
- 4. Betweenness centrality: Betweenness centrality (B(i)) describes the centrality of node i in the entire network, which is a parameter defined based on the perspective of network information flow. When B(i) is greater than 2.0, the region represented by node i is a hub region in the network^33^.

### Statistical analysis

Statistical analysis of differences in demographic characteristics between groups: Statistical significance between quantitative variables was assessed by X2 tests, with Yates's or Fisher's correction if necessary. Student's t-tests were performed to evaluate data that followed a normal distribution. Bonferroni correction was applied for multiple comparisons. Significant differences between groups were identified at P < 0.05. Numerical variables are expressed as the mean ± SD. Qualitative variables are described as the absolute values of individuals in distinct groups. Statistical analyses were performed using SPSS 25.0 (IBM Corp., Armonk, NY, USA).

Metabolism analysis was conducted with whole-brain voxelwise statistics to evaluate differences in whole-brain glucose metabolism between groups, and the statistical threshold was set as P < 0.001 (P-value after family-wise error correction (PFWE) < 0.05, number of effective thresholds (K) = 50 voxels). Only clusters with more than 50 voxels were considered statistically significant brain regions. xjView software (version 9.6, https://www.alive learn.net/xjView) was used to spatially locate the brain regions with local differences in cerebral glucose metabolism. The metabolic values of the whole brain were extracted by MarsBaR (version 0.44, http://marsbar.sourceforge.net/tutorial/index.html). Pearson correlation coefficients were calculated to determine the correlations between the metabolic values of regions with metabolic differences and clinical severity (Burke-Fahn-Marsden Dystonia Rating Scale movement (BFMDRS-M) score).

Nonparametric tests were used to determine the significance of differences in brain network parameters between the Meige syndrome group and the healthy control group: 1. Using different density thresholds, the normalized Cp γ, the normalized characteristic Lp λ, the small-world index σ, and the betweenness centrality B(i) were calculated for the two groups. 2. After repeated random grouping, the generation of a new network and the calculation of network parameters 1000 times, the different distributions of various network parameters under different thresholds between groups were obtained. 3. We used the 95^th^ percentile of the distribution as the critical value for one-tailed analysis to determine whether the null hypothesis had a probability of type I error of 0.05. If the null hypothesis was rejected, then the intergroup difference in the brain network parameters was considered significant^46^.

## Data Availability

The data collected for the study, including individual participant data and a data dictionary defining each field in the set, will be made available to others. Deidentified participant data, participant data with identifiers, a data dictionary, or other specified data set will be made available. The study protocol, statistical analysis plan, informed consent form, and other related documents will be available. Upon publication, these data will be available from the corresponding author, Ruen Liu, who can be contacted at the email address liuruen@pku.edu.cn. With investigator support and after approval of a proposal and with a signed data access agreement, data will be shared.
